# Parent Knowledge of Screening and Genetic Testing in Retinoblastoma

**DOI:** 10.1155/2020/3839792

**Published:** 2020-04-08

**Authors:** Wei Xiao, Xian Ji, Huijing Ye, Huiqi Zeng, Yang Gao, Rongxin Chen, Xiaoyun Chen, Yuxiang Mao, Huasheng Yang

**Affiliations:** State Key Laboratory of Ophthalmology, Zhongshan Ophthalmic Center, Sun Yat-Sen University, Guangzhou, China

## Abstract

**Purpose:**

To evaluate parent knowledge of screening and genetic testing for retinoblastoma and its potential correlation with demographics, clinical features, and socioeconomical factors.

**Methods:**

It was a cross-sectional study conducted at the ocular oncology unit of a tertiary ophthalmic center in Southern China. A face-to-face interview was administered to parents of retinoblastoma children during hospitalization. Parent knowledge was assessed using the sum score of a 7-item questionnaire. Demographics and socioeconomic status were collected from the interview, and clinical data were retrieved from the medical records.

**Results:**

A total of 126 parents of retinoblastoma children were included. Parents accurately answered 66.7% to 84.9% of each item in the questionnaire. Only 37 (29.4%) parents correctly answered all 7 questions. Parent knowledge was positively correlated with education, but it was not associated with patients' laterality, sex, or household income. Physicians and the Internet were the major sources of parental health-related information. During the median follow-up of 492 days, 13 (61.9%) of 21 patients in the full-score group without genetic testing at baseline actually conducted testing during follow-up compared to 29 of 67 (43.3%) in the less-than-full-score group (*P*=0.136).

**Conclusion:**

Overall parent knowledge about retinoblastoma screening and genetic testing was moderate. Higher education was associated with greater parent knowledge. Future studies should validate our findings in other populations, especially in those with different cultural background and healthcare systems.

## 1. Introduction

Retinoblastoma (Rb) is the most common intraocular malignancy among children. It is estimated that approximately 8,000 new cases are incident each year worldwide, with ∼90% residing in middle- and low-income countries [1]. With the recent advances in treatment techniques, such as intra-arterial and intravitreal chemotherapy, the goals of management have been transformed from life and globe preservation for keeping a lifelong good vision in developed countries [2, 3]. However, in most middle- and low-income countries, the likelihood of globe and vision preservation is still low [4]. Such gaps in prognosis between nations may attribute to multiple factors, from the variations in treatment facilities, differences in health care systems, and medical expertise, to the public awareness towards retinoblastoma [1, 3, 4]. Of them, inadequate parent awareness and knowledge may directly lead to a delayed visit and poor compliance with treatment and tumor surveillance.

Retinoblastoma was the first cancer that was described as a genetic disease, with ∼45% of all retinoblastoma cases being in the heritable form. Due to the extremely low incidence of retinoblastoma among general population, mass screening program is less cost-effective and infeasible. An alternative, but feasible strategy is to screen children with a high risk of developing retinoblastoma, including siblings and first- and second-degree relatives of the affected children, namely, “targeted screening.” Genetic testing of constitutional DNA enables precise clinical screening of relatives and the future offspring. Without genetic testing, all children at risk should undergo multiple examination under anaesthesia (EUA) in the first three years of life to detect small and easy-to-treat tumors. Based on this rationale, the guideline of screening children at risk for retinoblastoma was issued in 2017 by the American Association of Ophthalmic Oncologists and Pathologists (AAOOP) in conjunction with the American Association for Pediatric Ophthalmology and Strabismus (AAPOS) and the American Academy of Pediatrics [5]. In most less developed countries, genetic services have been developed during recent years [6], but genetic testing and screening for retinoblastoma have not been the standard of care yet, unlike the situations in industrialized countries [7]. Major tertiary treatment centers in China use random screening strategy and periodic review for surveillance of risky children. In such cases, the doctor-patient communication and parent knowledge and understanding of the disease are highly dependent for compliance to treatment and the overall prognosis.

The widespread use of mobile Internet and social media has changed the sources for parents seeking disease-related information. Mobile Internet, instant messaging, and other novel technologies have enabled parents' greater access to medical knowledge. However, new media might be a “double-edged sword”; its impact on patient knowledge may be positive but sometimes negative [8, 9]. In terms of retinoblastoma, information provided by the mass media may be incomplete, exaggerated, or even incorrect, probably causing misunderstandings to retinoblastoma among parents. In this context, we conducted this study to evaluate the current parent knowledge towards retinoblastoma, with an emphasis on the genetic testing and screening aspects of this childhood cancer. We further aimed to figure out the sources of health-related information. By doing so, we attempted to determine ways to conduct effective and targeted educational campaigns for children at risk of developing retinoblastoma.

## 2. Materials and Methods

### 2.1. Study Population

This cross-sectional survey was conducted in Zhongshan Ophthalmic Center, China, from December 2017 through December 2018. Our institute is one of the largest treatment centers for retinoblastoma in China, primarily receiving referrals from the Southern provinces. This study was conducted adhering to the tenets of the Declaration of Helsinki. The protocol was reviewed and approved by the hospital Institutional Review Board (2016KYPJ028). Informed consent was obtained from the parents of all enrolled children.

### 2.2. Data Collection

We designed a 7-item questionnaire in simplified Chinese based on the AAOOP consensus on screening children at risk for retinoblastoma [5]. The questionnaire, which covered major aspects of genetic testing and screening for retinoblastoma (Table 1), consisted of 7 closed questions. Each question had three choices: “yes,” “no,” and an additional “I don't know” option to prevent guessing. Scoring criteria for each question were as follows: −1 for an incorrect answer, 0 for “I don't know,” and 1 for a correct one. The sum score for all questions represented the interviewee's level of knowledge. Possible sum scores ranged from −7 to 7, with higher scores indicating greater parent knowledge. Internal consistency of the questionnaire was acceptable (Cronbach's coefficient *α* = 0.72). Face-to-face interviews were conducted by a trained researcher (HQZ) on the first day of patients' hospitalization. A brief introduction was given prior to survey with an emphasis on answering each question based on interviewees' current understanding rather than guessing. Socioeconomic characteristics, including education, household income, and medical insurance, were collected after each interview, and all clinical data were retrieved from the electronic medical records. All patients were followed up as of August 2019 to observe whether they accepted genetic testing subsequently.

### 2.3. Statistical Analysis

Continuous variables were expressed as mean ± standard deviation (SD) or median with interquartile range (IQR), depending on data distribution. Categorical variables were summarized as counts and percentages. Parent knowledge by subgroups (e.g., laterality, sex, and residence) was compared using Mann–Whitney *U* test or Kruskal–Wallis rank test. Bonferroni correction was applied for multiple comparisons. The hierarchical clustering method was applied to classify subgroups of all participants. Statistical analyses were performed using R (version 3.6.1). A *P* value <0.05 was considered statistically significant unless otherwise specified.

## 3. Results

### 3.1. Characteristics of Patients and Respondents

A total of 126 parent-child dyads were enrolled during the study period. As for the patients, 29 (23.0%) were bilaterally affected, 72 (57.1%) were boy, and 3 (2.4%) had a family history of retinoblastoma. Unilateral patients had significantly greater mean age at both diagnosis and survey than bilateral ones (at diagnosis: 32.4 ± 20.3 months vs. 15.3 ± 13.0 months, *P* < 0.001; at survey: 33.9 ± 20.6 months vs. 24.8 ± 22.3 months, *P*=0.050). No significant difference was found in gender distribution by laterality (*P*=0.647, Table 2). Almost half of bilateral children completed genetic testing prior to our survey compared with 25.8% of unilateral children (*P*=0.050). In terms of characteristics of interviewees, 68 (54.0%) respondents were mothers. Approximately, half of the respondents had less than a high school education (*n* = 61, 48.4%) and a half had low-level household income (<5000 RMB, *n* = 60, 47.7%, Table 2).

### 3.2. Profiles of Parent Knowledge on Retinoblastoma

For every single item in the questionnaire, 62.7% to 84.9% of all parents accurately responded each question (Table 2). The third question was correctly answered by most parents (84.9%),“whether regular examination for the contralateral eye was necessary after enucleation in unilateral retinoblastoma patients.” The median total score of all respondents was 5 (range: −2–7). Less than one-third parents (*n* = 37, 29.4%) correctly answered all 7 questions.

### 3.3. Correlation of Clinical, Demographic, and Socioeconomic Factors with Parent Knowledge

**Figure 1:**
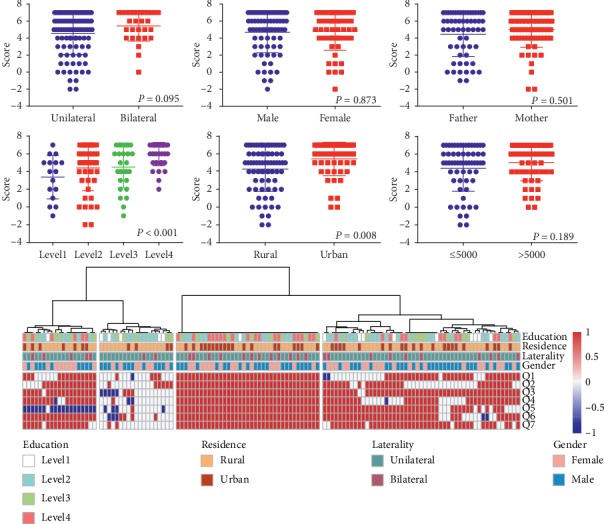
The associations between parent knowledge and multiple factors. The differences in median score between subgroups of laterality, sex, respondent, and household income are not statistically significant. Parents from urban areas are more knowledgeable than those from rural areas (*P*=0.008), but it is not significant after Bonferroni correction (adjusted *P* value = 0.0062). Median knowledge score is positively associated with educational attainment (*P* < 0.001). Unsupervised cluster analysis classifies all parents into four different subgroups. Note that the third group is with a higher level of education.

We compared the differences in parent knowledge by subcategories of clinical, demographic, and socioeconomic indexes. As shown in Figure 1, none of the differences of median score between laterality, sex, respondent (mother vs. father), and household income were significant (all *P* > 0.05). However, parents whose children living in urban areas were more knowledgeable than those from rural areas (*P*=0.008). Furthermore, median knowledge score was positively associated with educational attainment (*P* < 0.001). After Bonferroni correction for multiple comparisons (*P* threshold = 0.0062), only education attainment was associated with the total score (*P* < 0.001). Using the unsupervised cluster analysis, all parents were categorized into four different subgroups by knowledge. Parents in group A with the best knowledge had significantly higher level of education (Figure 1, *P* < 0.001).

### 3.4. Parent Knowledge and Actual Genetic Testing during Follow-Up

**Figure 2:**
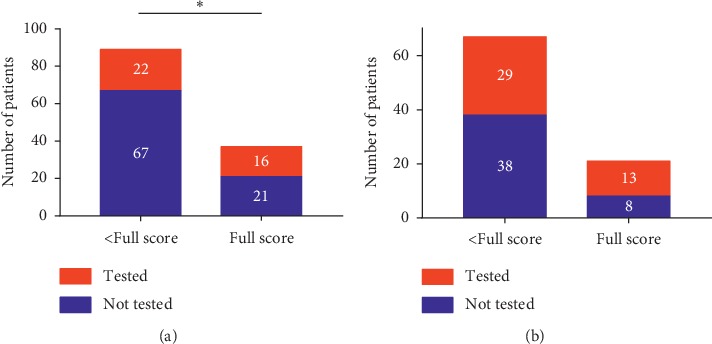
Parent knowledge and the action of genetic screening subsequently. At baseline, 16 (43.2%) children in full score group have completed genetic testing versus 22 (24.7%) in the less-than-full-score group (^*∗*^*P*=0.039). Over the median follow-up of 492 days (IQR: 403–586 days), 29 out of 67 (43.3%) children without genetic testing at baseline actually accepted testing inthe less-than-full-score group compared to 13 in 21 (61.9%) in the full-score group (*P*=0.136).

We then explored the association between parent knowledge and actual genetic testing during follow-up. At baseline, 16 (43.2%) children in the full-score group had completed genetic testing versus 22 (24.7%) in the less-than-full-score group (*P*=0.039). Over the median follow-up of 492 days (IQR: 403–586 days), 29 out of 67 (43.3%) children without genetic testing at baseline actually accepted testing inthe less-than-full-score group, compared to 13 in 21 (61.9%) in the full-score group (Figure 2, *P*=0.136).

### 3.5. Sources of Parent Knowledge

**Figure 3:**
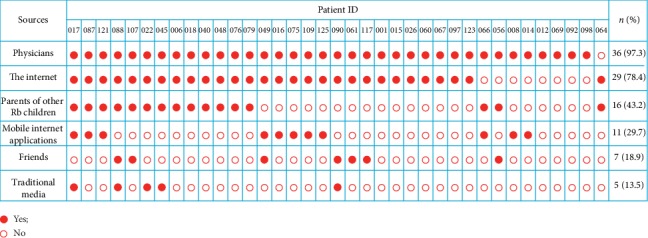
Sources of parent knowledge on retinoblastoma. Approximately, all parents (36 out of 37, 97.3%) gain knowledge from physicians. The second and third most frequent approaches are “the internet” (29/37, 78.4%) and “other retinoblastoma parents” (16/37, 43.2%), respectively. Mobile internet, friends, and classical media (e.g., newspaper and magazine) are less frequent ways to parent knowledge.

We analyzed the major sources through which the parents acquired the retinoblastoma-related information. We focused on the 37 full-scored parents. Approximately, all parents (36 out of 37, 97.3%) gained related knowledge from physicians including general practitioners, ophthalmologists, and ocular oncologists. The second and third most frequent approaches were “the Internet” (29/37, 78.4%) and “other retinoblastoma parents” (16/37, 43.2%), respectively (Figure 3). Mobile Internet, friends, and classical media (e.g. newspaper and magazine) were less frequent ways to parent knowledge.

## 4. Discussion

Better management of retinoblastoma partly relies on parents' understanding and knowledge of the disease, especially in areas and countries where the referral system and childhood cancer registry are less developed. In our study population, we found that less than one-third parents had adequate understanding to key but basic aspects of retinoblastoma genetic and clinical screening, implying an unmet need of health-related education for parents of retinoblastoma children.

Most published surveys have primarily focused on the knowledge of practitioners, rather than parents, and found that most first contact physicians lack sufficient knowledge of retinoblastoma [10]. A handful of studies on parental awareness have concentrated on the subset of familial retinoblastoma patients and consistently found that raised awareness improved early diagnosis and outcome [11, 12]. More recently, a qualitative study from Canada explored knowledge of retinoblastoma genetics among retinoblastoma survivors and parents of children with retinoblastoma, and it revealed that knowledge of retinoblastoma genetics was variable and often limited [13]. To our knowledge, this was the first study analyzing parent knowledge on retinoblastoma of both heritable and nonheritable forms.

Although there are many sources of information available currently, parents still lack sufficient disease awareness and knowledge in general. Our present study showed that parents' understanding of some critical issues was limited or even wrong. For example, over one-third of parents reported not knowing that mydriatic fundus examination was important for early diagnosis of retinoblastoma. Alarmingly, 35 parents (27.8%) responded that the siblings of the affected children standing a higher risk of developing retinoblastoma were wrong. With such misunderstanding, their parents might be unlikely to perform genetic or clinical screenings for at-risk children in their families, for example, the patient's siblings. Without timely diagnosis and screening and appropriate treatment, difficult-to-treat diseases may eventually develop in risky children.

Among the potentially related socioeconomic and clinical factors, only higher education was statistically associated with greater parent knowledge. It is easy to understand that well-educated parents may be good at seeking information from multiple sources. More importantly, they may be better at discriminating correct information compared with parents with lower educational attainment. Such positive correlation between parent education and disease-related knowledge was also documented in other studies, such as in sleep disorders [14]. In retinoblastoma management, our former study indicated that higher parental education was associated with a shorter lag time of treatment [15]. Taken together, these findings suggest that emphasis should be made on less-educated parents when carrying out retinoblastoma specific educational campaigns.

We unexpectedly found that none of the key factors, including laterality (bilateral or unilateral), timing of survey (initial or follow-up visit), respondent (mother or father), and household income, was associated with parent knowledge. It indicated that the impact of bilateral and unilateral key factors affected on parents' action to gain information on retinoblastoma was similar. It might be not necessary to consider the children's laterality when determining targeted population in education campaigns.

Parents usually have a strong willingness to obtain disease-related information through various pathways [16]. The Internet and mobile Internet have opened many new avenues for parents: they may diagnose retinoblastoma themselves online; share opinions on treatment; and form virtual communities to help optimize patient care. Despite the rapid growth of the Internet and mobile Internet has promoted the dissemination of medical knowledge among the public, the most common way through which parents got retinoblastoma information was still physician visits. It highlighted the significance of doctor-to-parent interactions in allaying uninformed fear and in reducing the rate of noncompliance with treatment. In this study, we also found that the Internet and communications with other parents also played important roles in knowledge acquirement. Owing to the high availability and accessibility of the Internet now, social movements could consider embracing social media as a means of spreading the aims and reaching wide audiences [17].

To our knowledge, this is the first quantitative study which investigated the current parent understanding of retinoblastoma. However, there were several limitations to be addressed. First, the time interval of diagnosis and survey was significantly longer in bilateral group than that in the unilateral group (Table 2). It made most bilateral patients interviewed at the time of follow-up rather than at the initial visit. This might have caused the difference in parent knowledge by laterality subgroup. Second, since our questionnaire was designed in Chinese and all interviewees were from mainland China, directly extrapolating the conclusion of our study to other population should be cautious due to the potential sociocultural differences. Third, some analysis might be limited by the small sample size in particular subgroups. For example, we did not find a correlation between full scores on the questionnaire and completion of genetic testing in follow-up (Figure 2). Such results might attribute to the small number of participants in the full score subcategory at baseline (*n* = 21), making the analysis insufficiently powered.

## 5. Conclusions

In conclusion, parent knowledge about retinoblastoma genetic testing and screening in South China was moderate. Parent knowledge was merely related to parents' education level but not related to child's gender, laterality, economic status, and timing of encounter. Health education on retinoblastoma should be deployed more on parents with low educational attainment.

## Figures and Tables

**Table 1:** Knowledge of retinoblastoma among parents.

Questions	Answers (*n*)			Correctly answered (*n*, %)
	Yes	No	Do not know	
Q1: Retinoblastoma can be inherited by the future offspring of the children	85	3	38	85 (67.5)
Q2: Mydriatic fundus examination is important for early diagnosis of retinoblastoma	84	0	42	84 (66.7)
Q3: For unilateral retinoblastoma patients, there is no need to regularly exam the contralateral eye if the affected eye was removed	5	107	14	107 (84.9)
Q4: The siblings of the affected children stand a higher risk of developing retinoblastoma	87	35	4	87 (69.0)
Q5: For children with higher risk, they should accept regular eye examination until they are elder than 3 years	22	79	25	79 (62.7)
Q6: Eye examination is essential for the siblings of bilateral Rb patients, but is not necessarily for siblings of unilateral patients	5	99	22	99 (78.6)
Q7: Genetic testing is useful in determining the risk of siblings and offspring	97	0	29	97 (77.0)

**Table 2:** Demographic and clinical features of retinoblastoma children and respondents by laterality.

	Laterality category			
	All (*n* = 126)	Unilateral (*n* = 97)	Bilateral (*n* = 29)	*P* value
Characteristics related to patients				
Age at diagnosis (months)	28.6 ± 20.2	32.4 ± 20.3	15.3 ± 13.0	<0.001
Age at survey (months)	31.9 ± 21.3	33.9 ± 20.6	24.8 ± 22.3	0.050
Genetic testing completed at survey (*n*, %)				
No	88 (69.8)	72 (72.2)	16 (55.2)	0.050
Yes	38 (30.2)	25 (25.8)	13 (44.8)	
Gender (*n*, %)				
Boy	72 (57.1)	57 (58.8)	15 (51.7)	0.647
Girl	54 (42.9)	40 (41.2)	14 (48.3)	

Characteristics related to respondents				
Respondent (*n*, %)				
Father	58 (46.0)	47 (48.5)	11 (37.9)	0.432
Mother	68 (54.0)	50 (51.5)	18 (62.1)	
Educational attainment				
Primary school or less	16 (12.7)	13 (13.4)	3 (10.3)	0.643
Junior high school	45 (35.7)	35 (36.1)	10 (34.5)	
High school	29 (23.0)	24 (24.7)	5 (17.3)	
College or higher	36 (28.6)	25 (25.8)	11 (37.9)	
Household monthly income, RMB				
<5000	60 (47.7)	48 (49.5)	12 (41.4)	0.733
≥5001	55 (43.6)	41 (42.3)	14 (48.3)	
Unknown	11 (8.7)	8 (8.2)	3 (10.3)	

## Data Availability

The data used to support the findings of this study are included within the article.
